# Transcription Regulatory Protein *SIN3* (YOL004W) Influences Mutation Rates in *Saccharomyces cerevisiae*

**DOI:** 10.17912/micropub.biology.001521

**Published:** 2025-02-26

**Authors:** Alexandra Eftimie, Damon Meyer

**Affiliations:** 1 School of Medicine, University of California, Riverside, Riverside, California, United States; 2 College of Health Sciences, California Northstate University, Rancho Cordova, California, United States

## Abstract

*SIN3* (YOL004W) codes for a protein in *Saccharomyces cerevisiae* which is suggested to function as a broad cellular transcription regulator through the binding of histone deacetylases and other enzymes to form a large protein complex that modifies chromatin. In addition, *SIN3* has also demonstrated potential roles in epigenetic silencing, DNA methylation, and centromere function. Here we report a new role of *SIN3* in affecting mutation rates within the *CAN1* reporter gene, suggesting an impact on genome stability.

**Figure 1. CAN1 forward mutation rate is significantly decreased in sin3Δ single mutants compared to wildtype:**
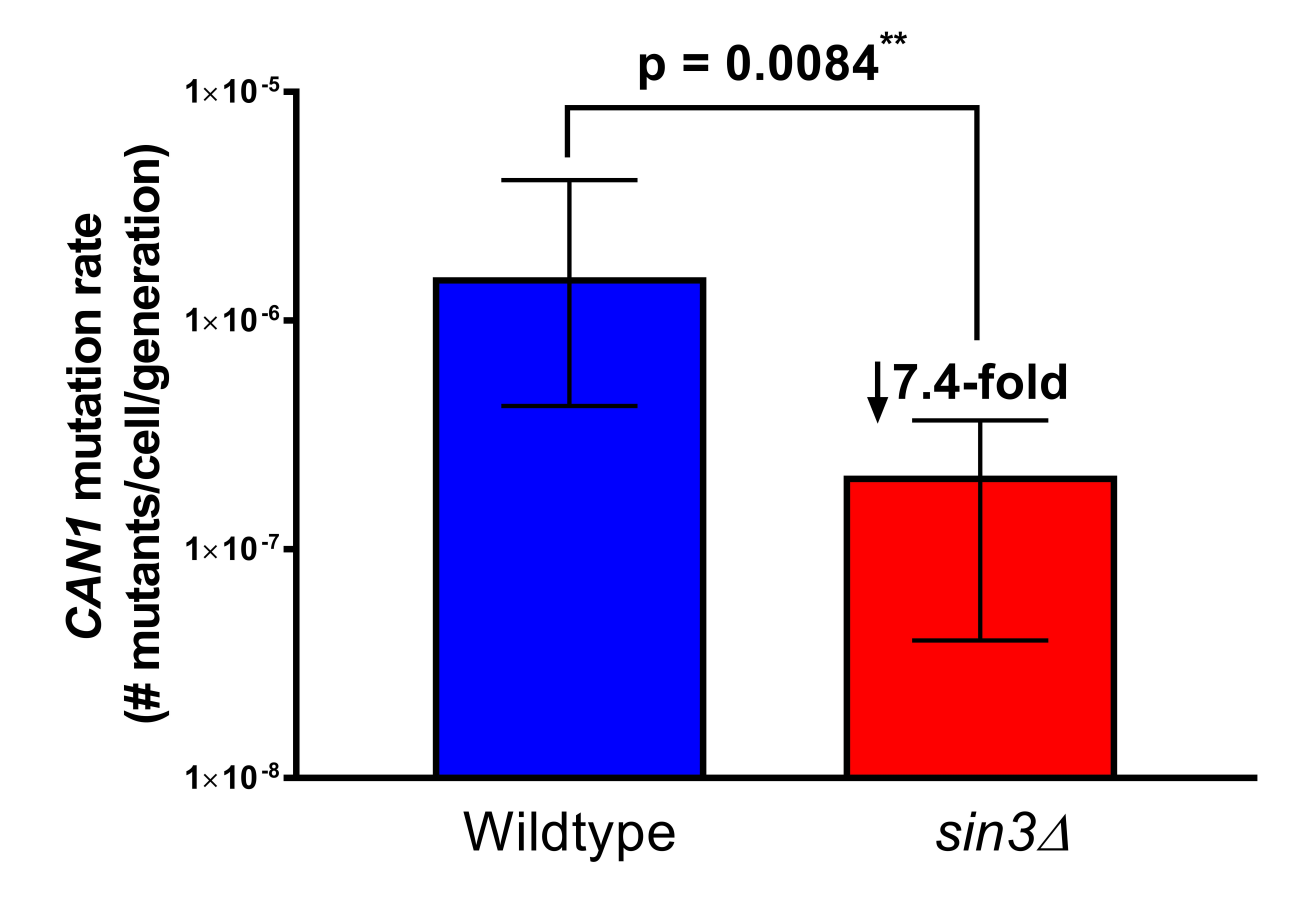
A minimum of 15 wildtype and *sin3Δ* single colonies were examined to determine the mutation rate within *CAN1* . The median, 95% confidence interval, and p-value following a student t-test were calculated. Fold difference between genotypes is shown.

## Description

Exploring the various functions of genes and uncharacterized open reading frames in the budding yeast, *S. cerevisia* e, is an important step in unraveling the biological processes that govern not only yeast but more complex organisms (Vanderwaeren et al., 2022; Bowling et al. 2016). However, while the function of many proteins within *S. cerevisiae* have been verified, the examination of uncharacterized or understudied genes in the literature has been declining (Tantoso et al., 2023). Related to this, discovery of new activities for gene products (RNA or protein) is difficult if the activity is not closely related to any known function of the RNA or protein. Therefore, we attempted to uncover genes within *S. cerevisiae* which may have a role in genome stability by screening the Saccharomyces Genome Database (SGD) for genes that are involved in DNA replication, meiosis, or decreased resistance to genotoxins. One such gene identified was *SIN3* , a conserved multifunctional regulator involved in chromatin modification and transcriptional regulation, exhibiting both corepressor and transcriptional stimulation capabilities through its association with a histone deacetylase complex (Silverstein & Ekwall, 2005; Adams et al., 2018). *SIN3* encodes a 175-kDa polypeptide which functions to negatively regulate the yeast HO gene, thereby determining the mating type pattern of *S. cerevisiae* , facilitate telomere folding structure, affects sensitivity to genotoxic agents, influence tri-nucleotide expansion, and a moderate sensitivity to UV radiation (Sternberg et al., 1987; Wang et al., 1990; Wagner et al., 2020; Westmoreland et al., 2009; Debacker et al. 2012; Hanway et al., 2002). These phenotypes suggest that Sin3 influences several processes within the cell, some of which impact DNA stability.

To further establish the biological function of Sin3 in maintaining genome stability, the *CAN1* forward mutation rate was evaluated in *sin3* Δ knockout mutants compared to wildtype (WT). The *CAN1* locus has been used as a proxy for genome mutation rates in *S. cerevisiae* since *can1* mutations are easily detected by colony growth on medium containing canavanine which also lacks arginine (Whelan et al., 1979). This includes examination of individual genes required for mutation accumulation and large-scale genome wide screens to identify previously unknown genes that contribute to mutations (Huang, et al., 2003; Novarina et al., 2020). We observed a significantly reduced spontaneous *CAN1* mutation rate of approximately 7.4-fold in *sin3* Δ knockout mutants in comparison to WT colonies, with the median colony counts being 2.1 x 10 ^-7^ and 1.55 x 10 ^-6^ respectively (p < 0.05). This data indicates that Sin3 plays a role in overall genome stability by influencing spontaneous mutation rates. However, it is not clear what known function of Sin3 may facilitate the increase in mutations. Previous evidence has shown a link between chromosome organization and genome landscape with varying mutation rates (Makova & Hardison, 2015). Sin3 is known to interact with histone deacetylase complexes, which influence gene expression patterns through histone modification and are typically deregulated in human cancers (Ropero & Esteller, 2007). In addition, histone deacetylase complex activity in humans and yeast have been demonstrated to favor trinucleotide repeat expansion while also promoting homology-directed repair (Lahue & Frizzell, 2012). This suggests that Sin3 may indirectly influence mutation rates through its role in chromosome modification and regulation of gene expression. Alternatively, we cannot rule out the possibility that Sin3 possesses a yet unknown function which more directly impacts mutation rates and thereby genome stability.

## Methods

*CAN1* Forward Mutation Assay

The *CAN1* mutation rate for wildtype and *sin3* Δ deletion mutations were determined from a minimum of fifteen individual freshly grown colonies for each genotype. Each colony was dispersed in 1 mL of sterile water and 0.2 mL was plated onto synthetic medium lacking arginine containing 60 ug/mL of canavanine. In addition, a dilution of the dispersed colony was plated onto YPD medium to determine the total viable cells within the colony. All cells plated were grown for 3 days at 30 ^ο^ C. The median rate of *CAN1* mutation was calculated by dividing the number of Can ^R^ colonies by the number of viable cells plated for each spore colony (Lea & Coulson, 1949). A 95% confidence interval was calculated for each genotype.

## Reagents

Yeast strains used in this study:

Number Genotype Source

ORF1 *MATa his3Δ1 leu2Δ0 met15Δ0 ura3Δ0* Horizon Discovery

ORF35 *MATa his3Δ1 leu2Δ0 met15Δ0 ura3Δ0 sin3::KANMX* Horizon Discovery
